# Tachysterol_2_ increases the synthesis of fibroblast growth factor 23 in bone cells

**DOI:** 10.3389/fnut.2022.948264

**Published:** 2022-07-25

**Authors:** Franz Ewendt, Julia Kotwan, Stefan Ploch, Martina Feger, Frank Hirche, Michael Föller, Gabriele I. Stangl

**Affiliations:** ^1^Institute of Agricultural and Nutritional Sciences, Martin Luther University Halle-Wittenberg, Halle (Saale), Germany; ^2^NutriCARD Competence Cluster for Nutrition and Cardiovascular Health, Halle (Saale), Germany; ^3^Department of Physiology, University of Hohenheim, Stuttgart, Germany

**Keywords:** UV-B irradiation, food fortification, photoisomer, vitamin D receptor, calcium, phosphate

## Abstract

Tachysterol_2_ (T_2_) is a photoisomer of the previtamin D_2_ found in UV-B-irradiated foods such as mushrooms or baker’s yeast. Due to its structural similarity to vitamin D, we hypothesized that T_2_ can affect vitamin D metabolism and in turn, fibroblast growth factor 23 (FGF23), a bone-derived phosphaturic hormone that is transcriptionally regulated by the vitamin D receptor (VDR). Initially, a mouse study was conducted to investigate the bioavailability of T_2_ and its impact on vitamin D metabolism and *Fgf23* expression. UMR106 and IDG-SW3 bone cell lines were used to elucidate the effect of T_2_ on FGF23 synthesis and the corresponding mechanisms. LC-MS/MS analysis found high concentrations of T_2_ in tissues and plasma of mice fed 4 vs. 0 mg/kg T_2_ for 2 weeks, accompanied by a significant decrease in plasma 1,25(OH)_2_D and increased renal *Cyp24a1* mRNA abundance. The *Fgf23* mRNA abundance in bones of mice fed T_2_ was moderately higher than that in control mice. The expression of Fgf23 strongly increased in UMR106 cells treated with T_2_. After *Vdr* silencing, the T_2_ effect on *Fgf23* diminished. This effect is presumably mediated by single-hydroxylated T_2_-derivatives, since siRNA-mediated silencing of *Cyp27a1*, but not *Cyp27b1*, resulted in a marked reduction in T_2_-induced *Fgf23* gene expression. To conclude, T_2_ is a potent regulator of Fgf23 synthesis in bone and activates Vdr. This effect depends, at least in part, on the action of Cyp27a1. The potential of oral T_2_ to modulate vitamin D metabolism and FGF23 synthesis raises questions about the safety of UV-B-treated foods.

## Introduction

Vitamin D insufficiency is highly prevalent worldwide due to seasonal variations in UV-B irradiation, extensive indoor activities that limit endogenous vitamin D synthesis, and a lack of vitamin D-rich foods (1). UV-B exposure of foods or food ingredients is a novel approach to increase the vitamin D concentration in food products and to prevent or combat vitamin D insufficiency (2). Recently, UV-B exposed baker’s yeast for the production of bread, rolls, or pastries (3) and mushrooms (4) have been approved by the European Food Safety Authority (EFSA) as novel foods to improve vitamin D status. Baker’s yeast and mushrooms have high quantities of ergosterol, which is converted to vitamin D_2_ after treatment with UV-B light (2, 5–8). However, UV-B exposure of ergosterol-rich foods leads to the formation of photoisomers such as tachysterol2 (T_2_) and lumisterol2 (L_2_) after the photoinduced ring opening of provitamin D_2_ (6, 9). The EFSA stated that T_2_ levels detected in UV-treated baker’s yeast or mushroom powder are low, and therefore are not harmful to health (3, 4). However, the toxicological assessment of the EFSA was not based on studies that investigated the potential biological effects of T_2_. Notably, recent data showed that orally administered L_2_ can enter the body of mice, decrease the circulating levels of 25-hydroxyvitamin D_3_ [25(OH)D_3_] and calcitriol, and affect genes encoding enzymes that are involved in vitamin D activation and catabolism (10). Thus, we hypothesized that T_2_ could also affect vitamin D metabolism and processes regulated by active vitamin D.

One hormone that is regulated by active vitamin D is fibroblast growth factor 23 (FGF23). FGF23 is an important regulator of phosphate homeostasis, which is predominantly synthesized in bone and secreted into the bloodstream (11). FGF23 primarily affects the kidney and suppresses renal phosphate reabsorption by internalizing and degrading the Na^+^-phosphate cotransporter type IIa (12). Additionally, FGF23 downregulates the expression of the 25-hydroxy vitamin D-1α-hydroxylase-encoding gene *CYP27B1* (13), the key enzyme for renal 1,25(OH)_2_D_3_ (active vitamin D) synthesis, together with its coreceptor α-Klotho (14).

FGF23 gained great clinical importance upon the discovery of its causal role in rare inherited diseases characterized by phosphate wasting and hypophosphataemic rickets (15). Elevated plasma concentrations of FGF23 are also associated with pathologies such as heart failure, cardiac hypertrophy, fibrosis, and dysfunction (16).

Thus, any factors that may modulate FGF23 production and secretion are of potential interest. Among endogenous factors (17), insulin (18), interleukin-6 (19), erythropoietin (20), tumor necrosis factor α (21), AMP-dependent protein kinase (22), myostatin (23), and cellular store-operated Ca^2+^ entry (24) have already been described as regulators of FGF23 in bone. Little is known about dietary factors that can modulate FGF23 synthesis.

The current study aimed to investigate the FGF23-modulating potential of oral T_2_ and the corresponding mechanism that may explain FGF23 changes.

## Materials and methods

### Animals and diets

Mice were cared for and handled according to the guidelines established by the National Research Council (25). The experimental procedures were approved by the committee for animal welfare of Martin Luther University Halle-Wittenberg (approval number: H1-4/T1-20). All mice were kept in pairs in a room controlled for temperature (22 ± 2°C), light (12 h light, 12 h dark cycle), and relative humidity (50–60%) and had free access to food and water.

To investigate, whether oral tachysterol_2_ (T_2_) can be absorbed and may influence vitamin D metabolism and FGF23 synthesis, eight 11–12-month-old male and female mice (Charles River, Sulzfeld, Germany) with an initial body weight of 32.7 ± 7.34 g were randomly allocated to two groups of four mice each and were fed basal diet without (control) or with 4 mg/kg T_2_ (Toronto Research Chemicals Inc., North York, ON, Canada) for 2 weeks. The chosen T_2_ dose represents the upper level of photoisomers in UVB-exposed food (26, 27). The basal diet consisted of (per kg) 388 g of starch, 200 g of sucrose, 200 g of casein, 100 g of soya oil, 60 g of a vitamin and mineral mixture, 50 g of cellulose, and 2 g of DL-methionine. With the exception of vitamin D, vitamins and minerals were added according to the recommendations of the US National Research Council (28). All mice had free access to their diets and water.

After the 2-week period, mice were deprived of food for 4 h and exsanguinated by decapitation. Plasma, intestinal mucosa, liver, and kidneys were obtained and stored at –80°C until the sterol and *Cyp* mRNA analysis. The femur and tibia were collected and removed from connective tissues, including both epiphyses and the bone marrow, and stored at –80°C until *Fgf23* mRNA analysis.

### Cell culture and treatments

UMR106 rat osteoblast-like cells (CRL-1661; ATCC, Manassas, VA, United States) were cultured in Dulbecco’s Modified Eagle Medium (DMEM) with high glucose supplemented with 10% fetal bovine serum (FBS), 100 U/ml penicillin, and 100 μg/ml streptomycin (all reagents from Gibco, Life Technologies, Darmstadt, Germany). For experiments, 2 × 10^5^ cells per 6-well were used. To increase the low basal *Fgf23* expression in these cells, UMR106 cells were pretreated with 10 nM 1,25(OH)_2_D_3_ (Tocris, Bristol, United Kingdom) for 24 h. Subsequently, the cells were treated with increasing concentrations of T_2_ (1–25 μM, Toronto Research Chemicals, Canada) for 24 h.

IDG-SW3 mouse osteocytes (CVCL_0P23; Kerafast, Boston, MA, United States) were cultured under proliferative conditions at 33°C and 5% CO_2_ in α-Minimum Essential Medium (Gibco, Life Technologies) supplemented with 10% FBS, 100 U/ml penicillin, and 100 μg/ml streptomycin, and 50 U/ml interferon-γ (IFN-γ) in rat tail type I collagen-coated cell culture flasks (all reagents from Gibco, Life Technologies). For experiments, cells were seeded on collagen-coated 12-well plates (1.5 × 10^5^ cells per well) and grown under proliferative conditions for 24 h. Differentiation was induced by removing IFN-γ from the medium and culturing the cells in differentiation medium containing 50 μg/ml ascorbic acid (Sigma-Aldrich, Schnelldorf, Germany) and 4 mM β-glycerophosphate (AppliChem, Darmstadt, Germany) at 37°C and 5% CO_2_. The medium was changed every 2nd to 3rd day. On day 27 of differentiation, cells were treated with 25 μM T_2_ or vehicle in duplicate for 24 h.

### Silencing

For silencing of *Vdr*, *Cyp27a1*, or *Cyp27b1*, 1.5 × 10^5^ UMR106 cells were seeded per well for 24 h in DMEM supplemented with 10% FBS, 100 U/ml penicillin, and 100 μg/ml streptomycin. Next, cells were transfected in antibiotic-free complete medium using 100 nM ON-TARGETplus Rat SMARTpool *Vdr* siRNA (L-097753-02-0020, Dharmacon, Lafayette, CO, United States), 100 nM ON-TARGETplus Rat SMARTpool *Cyp27a1* siRNA (L-093235-02-0005, Dharmacon), 100 nM ON-TARGETplus Rat SMARTpool *Cyp27b1* siRNA (L-091842-02-0005, Dharmacon), or 100 nM ON-TARGETplus non-targeting control siRNA (L-001810-10-20, Dharmacon), and DharmaFECT 1 transfection reagent (5 μl for *Vdr* siRNA transfection; 7.5 μl for *Cyp27a1* and *Cyp27b1* siRNA transfection; T-2001-02, Dharmacon). After 24 h of *Vdr* silencing and 72 h of *Cyp27a1* or *Cyp27b1* silencing, 10 nM 1,25(OH)_2_D_3_ was added to each well, and the cells were treated with 25 μM T_2_ or vehicle alone for another 24 h. In UMR106 cells treated with non-targeting siRNA, the relative *Vdr* expression was 1.28 ± 0.16 arbitrary units and 0.37 ± 0.04 in cells treated with *Vdr*-specific siRNA (*n* = 10; *p* < 0.01). In cells treated with *Cyp27a1*-specific siRNA, the relative *Cyp27a1* expression was 1.87 × 10^–5^ ± 3.37 × 10^–6^ arbitrary units and 3.98 × 10^–5^ ± 4.83 × 10^–6^ in control-treated cells (*n* = 6; *p* < 0.05). In cells treated with *Cyp27b1*-specific siRNA, the relative *Cyp27b1* expression was 0.0048 ± 0.0007 arbitrary units and 0.0097 ± 0.001 in cells treated with non-targeting siRNA (*n* = 6; *p* < 0.05).

### Analysis of D-vitamers in mice and cells

The concentration of T_2_ in plasma and tissues was analyzed by high-performance liquid chromatography (HPLC; 1260 Infinity Series, Agilent Technologies, Waldbronn, Germany) coupled to an electrospray ionization tandem mass spectrometer (MS/MS, QTRAP 5500, SCIEX, Darmstadt, Germany). Triple-deuterated vitamin D_3_ (Sigma-Aldrich, Munich, Germany) was added to the samples as an internal standard. Sample preparation and derivatization were done as described elsewhere (29). Tissues were dissolved in n-hexane/isopropanol (99:1 v/v), purified by normal-phase HPLC (1100 Series, Agilent Technologies) (30, 31), and then derivatized with PTAD (29). The derivatized samples were dissolved in methanol, mixed with a 10 mM ammonium formate solution (4:1, v/v, Sigma-Aldrich), and analyzed by LC–MS/MS. To quantify T_2_, the HPLC system was equipped with a Kinetex C18 column (100 A, 2.6 μm, 100 × 2.1 mm^2^, Phenomenex, Torrance, United States); the mobile phase consisted of (A) acetonitrile and (B) a mixture of acetonitrile/water (1:1, v/v) with 5 mM ammonium formate and 0.1% formic acid. The column temperature and gradient were as described elsewhere (32). Ionization for mass spectrometric analyses was induced by positive electrospray ionization, and data were recorded in multiple reaction monitoring (MRM) mode with the following transitions (quantifier ions) [M + PTAD + H + ]: T_2_, 572 > 395; vitamin D_3_-d_3_, 563 > 301. Mass transitions of T_2_ were verified by qualifier ions (T_2_, 572 > 377). Calibration curves were constructed using external standards of T_2_ (Toronto Research Chemicals, Canada) spiked with internal standards (as described above). The limit of quantification (LOQ) for plasma samples was 0.4 nM for T_2_ and 0.4 ng/g for tissue samples.

To determine the purity of the T_2_ used for the cell culture study, vitamin D_2_ was quantified by the same LC-MS/MS method described above (quantifier ions: vitamin D_2_, 572 > 298; qualifier ions: vitamin D_2_, 572 > 280), using a Poroshel 120 EC-C18 column (2.7 μm, 50 × 4.6 mm^2^, Agilent Technologies, Germany). Cell pellets and supernatant of UMR106 cells were prepared as plasma samples. Data show only traces of vitamin D_2_ in the T_2_ stock solution and T_2_-treated cells (2.09 ± 0.11 ng/mg). Additionally, the intracellular concentration of 25(OH)D_2_ in the T_2_-treated cells was below the LOQ of 0.025 ng/ml, assuming that the traces of vitamin D found in the T_2_ product were not responsible for the observed T_2_ effects.

### Analysis of minerals in plasma

The plasma concentrations of ionized calcium and inorganic phosphate were determined spectrophotometrically according to the manufacturer’s protocols (Calcium AS FS and Phosphate FS both from DiaSys Diagnostic Systems, Holzheim, Germany).

### Qualitative expression analysis

Untreated UMR106 rat osteoblast-like cells were used for total RNA extraction with TriFast reagent (Peqlab, Erlangen, Germany). cDNA synthesis was performed at 25°C for 5 min, 42°C for 1 h, and 70°C for 15 min using 1.2 μg of total RNA, the GoScript™ Reverse Transcription System, and random primers (both Promega, Mannheim, Germany). RT-PCR was conducted in a Rotor-Gene Q Cycler (Qiagen, Hilden, Germany) with 2 μl of cDNA (95°C for 3 min, 40 cycles of 95°C for 10 s, 58°C (*Cyp11a1*) or 60°C (*Cyp2r1*; *Cyp27a1*; *Cyp27b1*; *Cyp24a1*) for 30 s, and 72°C for 30 s). The primers used are listed in Table 1. Amplified RT-PCR products were loaded on a 1.5% agarose gel and visualized by Midori Green.

**TABLE 1:** Primers used for the analysis of the relative mRNA abundance of genes.

Gene	Species	Sequence F: 5′–3′ R: 5′–3′	Annealing [°C]	Accession number
*Alpl*	Rat	ACCTCTTAGGTCTCTTTGAG CTTTGGGATTCTTTGTCAGG	56	NM_013059.2
*Cyp2r1*	Rat	TTTCTCTAGGGAGAAGACAC ATATTTGTGCTCTTTCAGCG	60	NM_001108499.1
*Cyp27a1*	Rat	GAAGAGAGAGGACGATAACTC CTTTTGTATCAGCCTTGACAG	60	NM_178847.3
*Cyp11a1*	Rat	CTCCAGACTTATTTCGACTC GGTGTATTCATCAGCTTTACTG	58	NM_017286.3
*Cyp27b1* *Cyp27b1*	Rat Mouse	AGTGTTGAGATTGTACCCTG CGTATCTTGGGGAATTACATAG AGTGTTGAGATTGTACCCTG CGTATCTTGGGGAATTACATAG	60 58	NM_053763.1 NM_010009.2
*Cyp24a1*	Mouse	CGTTCTGGGTGAATACACGCTAC TTCGGGTCTAAACTTGTCAGCATC	58	NM_009996.4
*Cyp24a1*	Rat	AAAGAATCCATGAGGCTTAC TTTTCTCCTTTTGAAGCCAG	60	NM_201635.3
*Fgf23*	Rat	TAGAGCCTATTCAGACACTTC CATCAGGGCACTGTAGATAG	57	NM_130754.1
*Fgf23*	Mouse	TCGAAGGTTCCTTTGTATGGA AGTGATGCTTCTGCGACAAGT	58	NM_022657.4
*Galnt3*	Rat	TAGGGGGAAATCAGTACTTTG CTTTATAGACACATGCCTTCAG	60	NM_001015032.3
*Gapdh*	Mouse	GGTGAAGGTCGGTGTGAACG CTCGCTCCTGGAAGATGGTG	58	NM_001289726.1
*Spp1*	Rat	TGATGAACAGTATCCCGATG AACTGGGATGACCTTGATAG	60	NM_012881.2
*Tbp*	Mouse	CCAGACCCCACAACTCTTCC CAGTTGTCCGTGGCTCTCTT	60	NM_013684.3
*Tbp*	Rat	ACTCCTGCCACACCAGCC GGTCAAGTTTACAGCCAAGATTCA	57	NM_001004198.1
*Vdr*	Rat	CCTTTCTCCTGCCCAGCCTAACAC TCCCCCGGGTCAGAATAACACAG	64	NM_017058.2

Alpl, alkaline phosphatase; Cyp2r1, vitamin D 25-hydroxylase; Cyp27a1, cytochrome P450 27a1; Cyp11a1, cytochrome P450 11a1; Cyp27b1, 25-hydroxy vitamin D-1α-hydroxylase; Cyp24a1, 1,25(OH)_2_D_3_-24-hydroxylase; Fgf23, fibroblast growth factor 23; Galnt3, UDP-GalNAc:polypeptide N-acetylgalactosaminyltransferase 3; Gapdh, glyceraldehyde-3-phosphate dehydrogenase; Spp1, osteopontin; Tbp, TATA-box binding protein; Vdr, vitamin D receptor.

### RNA isolation and quantitative real-time PCR

The femurs and tibias used for total RNA extraction were ground in liquid nitrogen, transferred to TriFast reagent (Peqlab, Germany), and homogenized. Total RNA was extracted by adding chloroform and isolated by using the RNeasy Mini Kit (Qiagen, Germany). The RNA concentration was calculated using a NanoDrop Spectrophotometer (Thermo Fisher Scientific, Waltham, MA United States). Total bone RNA (300 ng) was reverse-transcribed (25°C for 5 min, 42°C for 1 h, and 70°C for 15 min) using the GoScript™ Reverse Transcription System (Promega, Germany).

Total kidney RNA was isolated with TriFast reagent (Peqlab, Germany) according to the manufacturer’s protocol, and cDNA was synthesized using M-MLV Reverse Transcriptase (Promega, Germany).

Total RNA was extracted from UMR106 osteoblast-like cells and IDG-SW3 osteocytes using TriFast reagent (Peqlab, Germany), and 1.2 μg was used for first-strand cDNA synthesis (25°C for 5 min, 42°C for 1 h, and 70°C for 15 min) with random primers and the GoScript™ Reverse Transcription System. Quantitative real-time PCR using a Rotor-Gene Q Cycler (Qiagen, Germany) and GoTaq qPCR Master Mix (Promega, Germany) was performed to determine relative *Fgf23*, *Galnt3*, *Spp1*, *Alpl*, *Vdr*, *Cyp27a1*, *Cyp27b1*, *Gapdh*, and *Tbp* expression. The qRT-PCR conditions were as follows: 95°C for 3 min; 40 cycles of 95°C for 10 s, 57°C for 30 s (rat *Fgf23* and rat *Tbp*) or 58°C for 30 s (mouse *Fgf23*, *Cyp27b1*, and *Gapdh*) or 60°C for 30 s (rat *Cyp27a1*, *Cyp27b1*, *Galnt3*, and *Spp1* or mouse *Tbp*) or 64°C for 30 s (rat *Vdr*) or 56°C for 30 s (rat *Alpl*), and 72°C for 30 s. The calculated mRNA expression levels of the examined genes were normalized to the expression levels of *Tbp* or *Gapdh*. The quantification of gene expression is presented as 2^−Δ*CT*^ (ΔC_T_ = C_T_ [target gene]—C_T_ [reference gene]) transformed data (33). The primers used are listed in Table 1.

### Enzyme-linked immunosorbent assay

UMR106 cells were cultured as described and treated with 25 μM T_2_ for 24 h. The cell culture supernatant was stored at –80°C. For quantification, cell culture supernatants were first concentrated using Vivaspin 6 centrifugal concentrators (Sartorius, Göttingen, Germany), and C-terminal and intact FGF23 protein were determined using enzyme-linked immunosorbent assay (ELISA) kit’s Mouse/Rat FGF-23 (Intact) and Mouse/Rat FGF-23 (C-Term) (both from Immutopics, San Clemente, CA, United States) according to the manufacturer’s protocol.

The plasma concentration of 1,25(OH)_2_D was analyzed by a commercial ELISA (Immunodiagnostic Systems, Frankfurt am Main, Germany). Analyses were performed by following the procedures given by the manufacturers with modifications described elsewhere (34).

### Western blotting

To determine nuclear VDR translocation, 2.1 × 10^6^ UMR106 cells were seeded in a 75-cm^2^ cell culture flask in complete medium for 24 h without 1,25(OH)_2_D_3_-prestimulation. Subsequently, the cells were treated with 25 μM T_2_ or vehicle alone for another 24 h, and then processed according to the protocol of the NE-PER kit (Thermo Fisher Scientific, United States). The obtained cell pellet was used for the extraction of cytoplasmic and nuclear proteins according to the manufacturer’s protocol. Next, 20 μg of cytoplasmic and nuclear protein was used for a standard western blot procedure using the following antibodies: VDR (D-6): sc-13133 (Santa Cruz Biotechnology, Dallas, TX, United States), GAPDH (#5174S), histone H3 (#9715S), and the secondary antibody anti-rabbit IgG (#7074) conjugated with HRP (all antibodies from Cell Signaling Technology, Frankfurt, Germany). Protein bands were visualized using ECL detection reagent (GE Healthcare-Amersham, Amersham, United Kingdom) and Syngene G:BOX Chemi XX6 (VWR, Dresden, Germany) documentation system. Protein band intensities of cytoplasmic VDR were normalized to GAPDH, and nuclear VDR band intensities were normalized to histone H3.

### Statistics

The data are shown as arithmetic means ± SEM, and *n* represents the number of independent experiments. Data were tested for normal distribution using the *Shapiro-Wilk* normality test. Two groups were tested for significant differences using an unpaired Student’s *t*-test (with Welch correction, if necessary) or a Mann-Whitney *U*-test (for non-normally distributed data). Data with more than two treatments were compared by one-way *ANOVA* followed by *Tukey’s* multiple comparison test (if necessary, *Welch’s ANOVA* followed by *Dunnett’s T3* multiple comparison test) or *Kruskal-Wallis* followed by *Dunn’s* multiple comparison test for non-normally distributed data. Differences were considered significant at *p* < 0.05.

## Results

### Oral tachysterol_2_ enters the body and affects vitamin D metabolism

**FIGURE 1:**
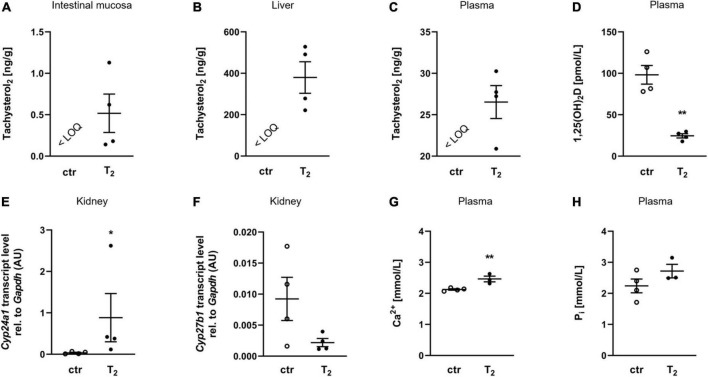
Oral tachysterol_2_ enters the body and affects vitamin D metabolism. Scatter dot plots and arithmetic means ± SEM of concentrations of tachysterol_2_ (T_2_) in the intestinal mucosa **(A)**, liver **(B)** and plasma **(C)**, plasma concentration of 1,25(OH)_2_D **(D)**, relative (rel.) renal *Cyp24a1* mRNA abundance (normalized to *Gapdh*) **(E)**, rel. renal *Cyp27b1* mRNA abundance (normalized to *Gapdh*) **(F)**, plasma concentration of ionized calcium (Ca^2+^) **(G)**, and plasma concentration of inorganic phosphate (P_i_) **(H)** of mice fed diets with 0 (ctr) or 4 mg/kg T_2_ for 2 weeks (**A–F**: *n* = 4). *p < 0.05 and **p < 0.01 indicate significant differences from the control. The limit of quantification (LOQ) for T_2_ was 0.4 nM in plasma and 0.4 ng/g in tissue samples. 1,25(OH)_2_D, active vitamin D, calcitriol; AU, arbitrary units; ctr, control [**D,F**: unpaired Student’s *t*-test with Welch’s correction; **(E)** Mann-Whitney *U*-test; **(G,H)** unpaired Student’s *t*-test (*n* = 3–4)].

The final body weights (33.5 ± 4.31 g for the control group and 38.48 ± 9.27 g for the T_2_ group) and feed intake per cage (3.33 ± 0.94 g/d for the control and 3.55 ± 0.72 g/d for the T_2_ group) did not differ between the two groups. To determine whether dietary T_2_ can enter the body, mice were fed T_2_ and analyzed for their concentrations in intestinal mucosa, liver, and plasma. T_2_ concentrations were found in the intestinal mucosa, liver, and plasma of mice that received 4 mg/kg T_2_ in their diet for 2 weeks, whereas no detectable T_2_ was observed in control mice (Figures 1A–C). Interestingly, the T_2_ group was characterized by a significant reduction in plasma 1,25(OH)_2_D levels (Figure 1D) and a marked increase in the mRNA abundance of *Cyp24a1* in the kidneys compared to the control group (Figure 1E). Additionally, the mRNA abundance of renal *Cyp27b1* was moderately but not statistically significantly lower in the T_2_ group than in the control group (Figure 1F). Interestingly, despite reduced levels of 1,25(OH)_2_D, the T_2_ group had significantly higher plasma concentrations of ionized calcium (Figure 1G) and a trend toward higher plasma levels of inorganic phosphate than the control group (Figure 1H).

### Tachysterol_2_ induces fibroblast growth factor 23 production in bone cells

**FIGURE 2:**
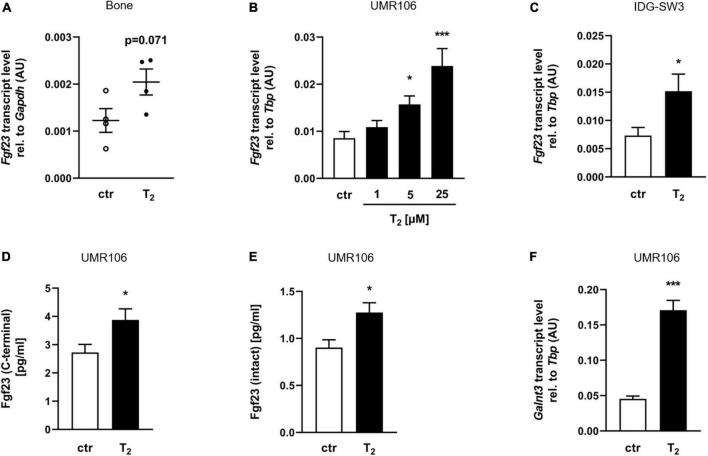
Tachysterol_2_ induces Fgf23 production in bone cells. **(A)** Scatter dot plots and arithmetic means ± SEM of relative (rel.) *Fgf23* mRNA abundance (normalized to *Gapdh*) in bone of wild-type mice (*n* = 4) receiving diets with 0 or 4 mg/kg tachysterol_2_ (T_2_) for 2 weeks. Arithmetic means ± SEM of rel. *Fgf23* (**B** *n* = 8; **C** *n* = 11) and *Galnt3* (**F** *n* = 5) mRNA abundance (normalized to *Tbp*) or cell culture supernatant concentrations of C-terminal (**D** *n* = 10) or intact Fgf23 protein (**E** *n* = 11) in UMR106 osteoblast-like cells **(B,D,E,F)** and IDG-SW3 osteocytes **(C)** treated with T_2_ (**B** indicated concentrations; **C–F** 25 μM T_2_) for 24 h. *p < 0.05 and ***p < 0.001 indicate significant differences from the control. AU, arbitrary units; ctr, control (**A**,**D**,**E** unpaired Student’s *t*-test; **B** *Kruskal-Wallis*; **C,F** unpaired Student’s *t*-test with Welch’s correction).

Since oral T_2_ was absorbed by mice and affected vitamin D metabolism, the mRNA abundance of *Fgf23* was analyzed in the mouse bones, the major site of FGF23 synthesis. Interestingly, the femurs and tibias of mice fed T_2_ had moderately, but not statistically significantly higher relative transcript levels of *Fgf23* than those of the control group (Figure 2A) (*p* = 0.071). To investigate whether T_2_ can directly stimulate *Fgf23* mRNA expression, rat UMR106 osteoblast-like cells were treated with increasing concentrations of T_2_ for 24 h. Figure 2B illustrates that T_2_ increased *Fgf23* gene expression in a dose-dependent manner. Additionally, IDG-SW3 osteocytes treated with 25 μM T_2_ for 24 h showed a significant increase in *Fgf23* transcript levels (Figure 2C). The stimulated transcription of *Fgf23* in T_2_-treated UMR106 cells was accompanied by a significant release of C-terminal Fgf23 protein, whose measurement was based on a C-terminal assay for FGF23 which detects both intact FGF23 and biologically inactive C-terminal fragments (Figure 2D). Subsequent analyses showed that the increase in C-terminal Fgf23 was associated with an increase in intact Fgf23 protein in the supernatant of T_2_-treated UMR106 cells (Figure 2E). Because secretion of intact FGF23 depends on the catalytic action of GALNT3 (35), mRNA abundance of *Galnt3* in UMR106 cells was analyzed. Figure 2F demonstrates that the *Galnt3* mRNA concentrations of cells treated with 25 μM T_2_ for 24 h increased more than threefold compared to control cells.

### The effect of tachysterol_2_ on fibroblast growth factor 23 expression depends, at least in part, on regulation of the vitamin D receptor

**FIGURE 3:**
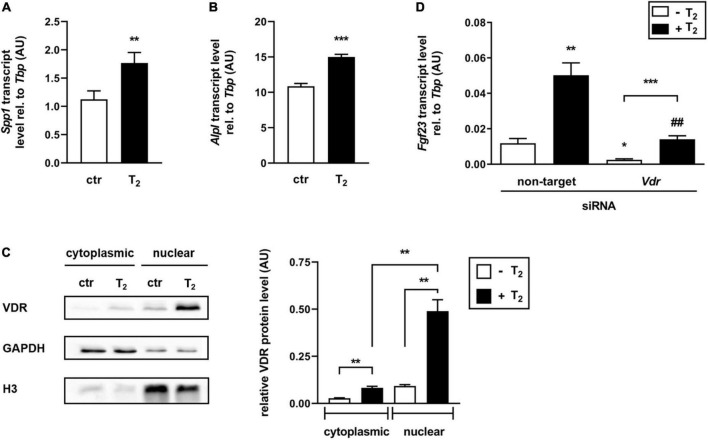
The effect of tachysterol_2_ on *Fgf23* expression depends, at least in part, on regulation of the vitamin D receptor. Arithmetic means ± SEM of relative (rel.) *Spp1* (**A** *n* = 6) or *Alpl* (**B** *n* = 5) mRNA abundance (normalized to *Tbp*) in UMR106 cells treated with 25 μM tachysterol_2_ (T_2_) for 24 h. **(C)** Original Western blots (left panel) showing cytoplasmic and nuclear VDR, GAPDH, and histone H3 (H3) protein abundance in UMR106 cells treated without (ctr) or with T_2_ (25 μM, 24 h). The right panel depicts the densitometric analysis (arithmetic mean ± SEM, *n* = 6) of cytoplasmic VDR (normalized to loading control GAPDH) and nuclear VDR (normalized to loading control histone H3) abundance in UMR106 cells treated without (white bars) or with (black bars) T_2_ (25 μM, 24 h). **(D)** Arithmetic means ± SEM of rel. *Fgf23* mRNA abundance (normalized to *Tbp*) in UMR106 cells incubated without (white bars) or with (black bars) T_2_ (25 μM, 24 h) in the presence or absence of non-targeting or *Vdr*-specific siRNA (100 nM, 48 h, *n* = 10). Treatment with *Vdr*-specific siRNA resulted in a 71% reduction of relative *Vdr* mRNA **(D)** expression compared to non-targeting siRNA as control. *p < 0.05, **p < 0.01, and ***p < 0.001 indicate significant differences from control. ^##^*p* < 0.01 indicate a significant difference from the absence of *Vdr*-specific siRNA (2nd vs. 4th bar). AU, arbitrary units; ctr, control [**A** Mann-Whitney *U*-test; **B** unpaired Student’s *t*-test; **C**,**D** Welch’s *ANOVA*].

Assuming that T_2_ may function as a vitamin D receptor (VDR) ligand, downstream targets of 1,25(OH)_2_D-VDR signaling were analyzed in UMR106 cells treated with 25 μM T_2_ for 24 h. Interestingly, T_2_ significantly induced gene expression of both *Spp1* (Figure 3A) and *Alpl* (Figure 3B) compared to the vehicle control, indicating that T_2_ can activate VDR. To investigate the role of VDR in mediating the T_2_ effect, Western blot analysis examining the translocation of VDR from the cytoplasm to the nucleus was conducted. As shown in Figure 3C, treatment of UMR106 cells with 25 μM T_2_ for 24 h resulted in a pronounced translocation of cytosolic Vdr into the nucleus compared to the control. In addition, T_2_ treatment significantly increased Vdr protein expression in the cytoplasm compared to the vehicle control (Figure 3C). To determine the involvement of VDR in T_2_-mediated regulation of *Fgf23* gene expression, T_2_ effects on *Fgf23* expression in UMR106 cells were elucidated in the presence or absence of *Vdr*-specific siRNA. Figure 3D demonstrates that *Vdr*-specific siRNA treatment significantly diminished T_2_-mediated upregulation of *Fgf23* gene expression. This effect suggests that *Vdr* is, at least in part, needed for T_2_ to regulate FGF23 production. Nevertheless, T_2_ was able to significantly increase *Fgf23* gene expression even in the presence of *Vdr*-specific siRNA, suggesting that T_2_ may also regulate *Fgf23* gene expression through other pathways.

### The effect of tachysterol_2_ on fibroblast growth factor 23 transcription is at least partially dependent on Cyp27a1

**FIGURE 4:**
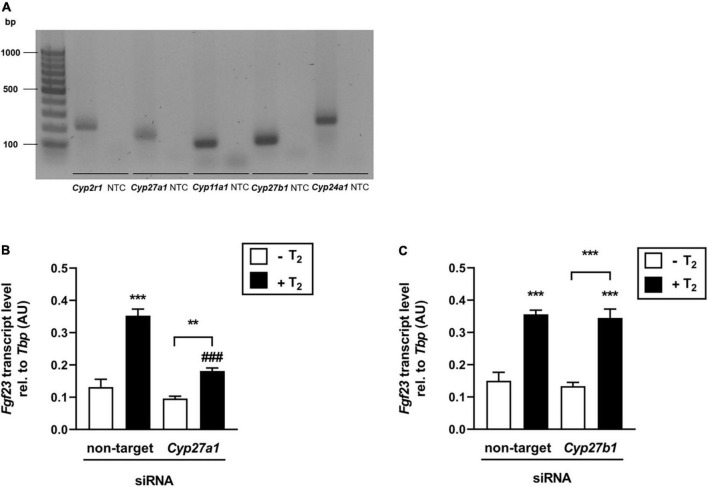
The effect of tachysterol_2_ on *Fgf23* transcription is at least partially dependent on Cyp27a1. **(A)** Original agarose gel photograph showing amplified *Cyp2r1*-, *Cyp27a1*-, *Cyp11a1*-, *Cyp27b1*- or *Cyp24a1*-specific cDNA in untreated UMR106 cells. **(B,C)** Arithmetic means ± SEM of relative (rel.) *Fgf23* mRNA abundance (normalized to *Tbp*) in UMR106 cells incubated with non-targeting or *Cyp27a1*-specific (**B**, 100 nM, 96 h, *n* = 6) or *Cyp27b1*-specific (**C**, 100 nM, 96 h, *n* = 6) siRNA in the absence (white bars) or presence (black bars) of T_2_ (25 μM, 24 h). Treatment with *Cyp27a1*-specific or *Cyp27b1*-specific siRNA resulted in a 53% reduction of relative *Cyp27a1* mRNA **(B,C)** 50% reduction of relative *Cyp27b1* mRNA expression compared to non-targeting siRNA as control. **p < 0.01 and ***p < 0.001 indicate significant differences from control. ^###^*p* < 0.001 indicates a significant difference from the absence of *Cyp27a1*-specific siRNA (2nd vs. 4th bar). bp, base pairs; NTC, non-template control; AU, arbitrary units (**B,C** one-way *ANOVA*).

The effect of T_2_ on *Fgf23* expression appears to be mediated by Vdr. Since non-hydroxylated vitamin D photoisomers are suggested to have a low binding affinity to Vdr in contrast to hydroxylated compounds (36), we hypothesized that T_2_ is metabolized by cytochrome P450 (CYP) enzymes to form active T_2_ derivates, thereby enhancing their effect on the Vdr-mediated regulation of *Fgf23* gene expression. Therefore, we first investigated whether bone cells express CYPs, which are involved in the hydroxylation of vitamin D metabolites. Figure 4A shows that *Cyp2r1*, *Cyp27a1*, *Cyp11a1*, *Cyp27b1*, and *Cyp24a1* were expressed in UMR106 cells, suggesting that T_2_ can be converted to hydroxy-T_2_ derivates. To elucidate the role of Cyp27a1 and Cyp27b1 in the production of bioactive T_2_ derivatives, which can stimulate FGF23 expression, we conducted two experiments using *Cyp27a1*- and *Cyp27b1*-specific siRNAs. Figure 4B illustrates that T_2_ significantly stimulated *Fgf23* gene expression in the presence of the non-targeting siRNA, whereas the effect of T_2_ on *Fgf23* expression was markedly lower in the presence of *Cyp27a1*-specific siRNA, indicating that the enzymatic activity of Cyp27a1 is, at least in part, necessary to mediate the T_2_ effect on *Fgf23* gene expression in UMR106 cells. In contrast, Cyp27b1 does not appear to be necessary to mediate the T_2_ effect on *Fgf23* expression, as *Fgf23* gene expression did not differ in cells treated with non-targeting or *Cyp27b1*-specific siRNA after T_2_ treatment (Figure 4C).

## Discussion

UV-B irradiation of baker’s yeast and mushrooms with high ergosterol content is used to enrich vitamin D_2_ content, making them alternative plant- and fungus-based vitamin D sources (2, 3, 5, 9, 37, 38). However, during UV-B irradiation and vitamin D_2_ synthesis, the photoproducts T_2_ and L_2_ are also generated (9, 27). The photoisomers tachysterol_3_ (T_3_) and lumisterol_3_ (L_3_) are also formed in human skin during the conversion of 7-dehydrocholesterol to vitamin D_3_, but their entry into the circulation is considered negligible (39). There are only a few studies addressing the absorption, metabolism or biological activity of photoisomers from food in mammals (6). In the scientific assessment conducted by the EFSA, tachysterol was not included in the product specification and safety due to the low concentrations of tachysterol in the consumable bread product which included UV-B-irradiated baker’s yeast (3).

Notably, this study provides novel evidence that orally administered T_2_ can be absorbed and affect vitamin D metabolism in mice and potentially stimulates the synthesis of the phosphaturic hormone FGF23 in bone cells.

The apparent intestinal absorption of oral T_2_, shown by considerable increases in T_2_ in the circulation and tissues of mice, is consistent with data showing that L_2_, another photoisomer produced by UV-B exposure of foods (26, 27), can enter the body after oral intake (10). Interestingly, the observed reduction in plasma 1,25(OH)_2_D levels, the increased mRNA abundance of renal *Cyp24a1* and the trend toward lower mRNA abundance of renal *Cyp27b1* in mice fed 4 mg/kg T_2_ demonstrates the potential of T_2_ to affect vitamin D metabolism in a way similar to the recently found effects of L_2_ (10). Based on the current data that indicate an up-regulation of *Cyp24a1*, the key enzyme responsible for the catabolism of active vitamin D, and a down-regulation of *Cyp27b1* which hydroxylates 25(OH)D to 1,25(OH)_2_D (40, 41), it is tempting to speculate that the observed decrease in plasma 1,25(OH)_2_D levels in T_2_-fed mice is the result of an accelerated degradation and decreased synthesis of this hormone. In addition, the higher calcium concentration in the plasma of T_2_-fed mice in comparison to control mice, suggests that T_2_ has calcitriol-like effects, which can compensate for the reduced 1,25(OH)_2_D levels.

1,25(OH)_2_D is a systemic stimulator of FGF23 synthesis mainly in bone (42). Because 1,25(OH)_2_D was affected by T_2_, we hypothesized that the mRNA abundance of *Fgf23* in the femur and tibia of T_2_-supplemented mice could have changed. Unexpectedly, mice fed T_2_ had moderately higher *Fgf23* transcript levels (*p* = 0.071) in their bones than the control group, despite their marked reduction in circulating 1,25(OH)_2_D. These findings indicate that T_2_ or T_2_-metabolites are likely to have direct effects on *Fgf23* gene expression in bone, which are independent of the 1,25(OH)_2_D action. This hypothesis was tested using UMR106 osteoblast-like cells that were treated with increasing concentrations of T_2_ and IDG-SW3 osteocytes. The data further show very impressively that T_2_ can stimulate the gene expression of *Fgf23*, and in turn the subsequent release of intact Fgf23 protein by *Galnt3*, the catalytic enzyme which is required for secretion of intact FGF23 (35).

Because T_3_, which is formed in skin *via* UV-B exposure (39), has been shown to bind to VDR albeit with low affinity (36), it was hypothesized that T_2_ could have mediated its stimulatory effect on FGF23 by acting as a vitamin D receptor agonist. The increase in the mRNA abundance of downstream targets of 1,25(OH)_2_D-VDR, namely, the *Spp1* and *Alpl* genes, indicated VDR-mediated action of T_2_. Moreover, T_2_ strongly induced Vdr translocation into the nucleus, indicating a potential binding and activation of the transcription factor by T_2_. T_2_ also induced increased intracellular Vdr protein levels. This phenomenon is also reported for 1,25(OH)_2_D which induces not only the translocation of VDR from cytosol to the nucleus but also the expression of VDR, the stabilization of *VDR* mRNA and the protection of VDR protein from degradation (43). It is therefore likely that T_2_ had increased the intracellular Vdr levels by similar mechanisms as those described for 1,25(OH)_2_D. Additionally, the siRNA-mediated knockdown of *Vdr* in UMR106 cells, weakened the effect of T_2_ on *Fgf23* gene expression. Thus, it can be concluded that T_2_ stimulates FGF23 synthesis, at least in part, *via* the activation of VDR. This mechanism is similar to the well-known effect of 1,25(OH)_2_D, which stimulates *Fgf23* expression *via* the VDR and binding at vitamin D responsive element (VDRE) in the promotor region of FGF23 (44). However, *Fgf23* gene expression was higher in cells treated with both *Vdr*-specific siRNA and T_2_ than in cells treated with *Vdr*-specific siRNA alone, suggesting that T_2_ may also act through other signaling pathways. Considering that protein kinase C (PKC) induces *Fgf23* gene expression in UMR106 cells (45) and that 1,25(OH)_2_D_3_ has also been shown to stimulate PKC in these cells (46), it is tempting to speculate that T_2_ or T_2_-metabolites, similar to 1,25(OH)_2_D_3_, may mediate non-genomic (VDRE-independent) activation of PKC that stimulates *Fgf23* gene expression in a VDR-independent manner.

Next, we were interested in the bioactive form of T_2_ and the question of whether non-hydroxylated or hydroxylated T_2_ derivatives were responsible for the stimulated FGF23 synthesis. We hypothesized that hydroxylated T_2_ would have a greater potential to stimulate FGF23 than native T_2_, because published data on T_3_ found a 1000-fold higher binding affinity of 25-hydroxylated T_3_ derivatives than of non-hydroxylated compounds (36). Different types of cells, such as enterocytes, hepatocytes, keratinocytes, and kidney cells, express cytochrome P450 (CYP) enzymes, which are necessary for the hydroxylation of D vitamers (40). Here, we found that UMR106 cells can also express these types of enzymes, suggesting that T_2_ can be converted to hydroxy-T_2_ derivatives. To elucidate whether enzymes mediating 25-hydroxylation or 1,25-hydroxylation are crucial for the bioactivity of T_2_, siRNA-mediated knockdown of Cyp27a1 and Cyp27b1 was induced in UMR106 cells. Unexpectedly, we found that Cyp27a1 but not Cyp27b1 was necessary to synthesize T_2_ derivatives that stimulate *Fgf23* expression. Our results suggest that T_2_ is metabolized in UMR106 cells and that hydroxylation of T_2_ modulates its biological activity, although 25-hydroxylation of T_2_ *via* Cyp27a1 appears to be more important than metabolism *via* Cyp27b1 for this purpose. This means that CYP27B1, which is usually pivotal to synthesizing the VDR-stimulating vitamin D hormone (40), does not play a crucial role in the generation of bioactive T_2_. The current findings corroborate data that showed that synthetic dihydrotachysterol_3_ (DHT_3_), a synthetic reduction product of T_3_, is less effective in the bone mobilization of minerals to elevate plasma Ca^2+^ than synthetic 25-hydroxy-DHT_3_ [25(OH)DHT_3_] (47). In addition, 25(OH)DHT_3_ was shown to be only 2.5 and 25 times less active than 1,25(OH)_2_DHT_3_ or 1,25(OH)_2_D_3_, respectively, in inducing reporter gene expression in COS-1 cells transfected with a VDRE-GH reporter expression system (48). Notably, *Fgf23* gene expression was higher in cells treated with both T_2_ and *Cyp27a1*-specific siRNA than in cells treated with *Cyp27a1*-specific siRNA alone. These findings indicate of the involvement of other 25-hydroxy enzymes, such as CYP2R1 or CYP11A1 (40, 49), which, according to our results, are also expressed in UMR106 cells and thus may also be involved in the metabolization of T_2_.

In the safety assessment of UV-B irradiated foods such as baker’s yeast or mushrooms, the focus is primarily on the amounts of D_2_ formed, but the equally detectable, albeit smaller, amounts of tachysterol in these foods have thus far been neglected for this purpose (3, 4). In view of the steady increase in the worldwide consumption of mushrooms (50) and thus the possibly increased consumption of UV-irradiated mushrooms, the novel results of this study give reason to reconsider this assessment. Strikingly, our data show that orally administered photoisomer T_2_ enters the body, becomes biologically active, and interferes with vitamin D metabolism in mice, which is consistent with results from a previous study of the photoisomer L_2_ (10). Furthermore, our experiments show the importance of the metabolism and resulting activation of T_2_ as a substrate of CYP enzymes, which are also central for the hydroxylation of D vitamers. Notably, this study showed that T_2_ stimulated the synthesis of biologically active Fgf23 in bone cells. There is increasing evidence of pathological effects of dysregulated FGF23 levels, such as induction of left ventricular hypertrophy, cardiac fibrosis, and dysfunction, and FGF23 is therefore discussed as a risk factor and biomarker for cardiovascular disease (16). In view of our findings, the consumption safety of UV-irradiated foods may need to be reconsidered due to the formation during manufacture of photoisomers such as T_2_ which show absorption and metabolism to potentially active metabolites.

To conclude, oral T_2_ can enter the body and is a potent regulator of Fgf23 production in bone cells. Based on the current findings, the effect of T_2_ appears to be mediated largely *via* Vdr and requires, at least in part, the metabolism of T_2_ by Cyp27a1. Due to the potential of T_2_ to induce 1,25(OH)_2_D degradation, activate VDR, and increase FGF23, which is associated with cardiovascular diseases, the safety of UV-B-treated foods is questionable.

## Data availability statement

The raw data supporting the conclusions of this article will be made available by the authors, without undue reservation.

## Ethics statement

The animal study was reviewed and approved by the Committee for Animal Welfare of Martin Luther University Halle-Wittenberg (approval number: H1-4/T1-20).

## Author contributions

FE: conceptualization, formal analysis, investigation, methodology, visualization, project administration, and writing—original draft, review and editing. JK and FH: formal analysis, methodology, and investigation. SP: investigation. MFe: formal analysis. MFö: supervision, resources, and writing—review and editing. GS: conceptualization, resources, funding acquisition, project administration, supervision, and writing—original draft, review and editing. All authors contributed to the article and approved the submitted version.
